# Clinical significance of CCR7^+^CD8^+^ T cells in kidney transplant recipients with allograft rejection

**DOI:** 10.1038/s41598-018-27141-6

**Published:** 2018-06-11

**Authors:** Kyoung Woon Kim, Bo-Mi Kim, Kyoung Chan Doh, Mi-La Cho, Chul Woo Yang, Byung Ha Chung

**Affiliations:** 1 Convergent Research Consortium for Immunologic disease, The Catholic University of Korea, Seoul, Korea; 2 Transplant research center, The Catholic University of Korea, Seoul, Korea; 3 Division of Nephrology, Department of Internal Medicine, Seoul St. Mary’s Hospital, College of Medicine, The Catholic University of Korea, Seoul, Korea

## Abstract

The regulatory function of CCR7^+^CD8^+^ T cells against effector T-cells involved in T-cell mediated rejection (TCMR) in kidney transplant recipients was investigated. *In vitro* experiments explored the ability of CCR7^+^CD8^+^ T cells to suppress T-cell proliferation under T-cell activation conditions or during coculture with human renal proximal tubular epithelial cells (HRPTEpiC). In an *ex vivo* experiment, the proportion of CCR7^+^/CD8^+^, FOXP3^+^/CCR7^+^CD8^+^ T and effector T-cell subsets were compared between the normal biopsy control (NC, n = 17) and TCMR group (n = 17). The CCR7^+^CD8^+^ T cells significantly suppressed the proliferation of CD4^+^ T cells and significantly decreased the proportion of IFN-γ^+^ and IL-17^+^/CD4^+^ T cells and inflammatory cytokine levels (all p < 0.05). After coculturing with HRPTEpiC, CCR7^+^CD8^+^ T cells also suppressed T-cell differentiation into IL-2^+^, IFN-γ^+^, and IL-17^+^/CD4^+^ T cells (all p < 0.05). The TCMR group had significantly fewer CCR7^+^/CD8^+^ and FOXP3^+^/CCR7^+^CD8^+^ T in comparison with the NC group, but the proportions of all three effector T-cell subsets were increased in the TCMR group (all p < 0.05). The proportion of CCR7^+^/CD8^+^ T was inversely correlated with those of effector T-cell subsets. The results indicate that CCR7^+^CD8^+^ T cells may regulate effector T-cells involved in TCMR in an *in vitro* and in an *ex vivo* transplant model.

## Introduction

Regulatory T cells (Treg) have been recognized as a specialized subset of T cells that participate in normal and dysfunctional immune responses^1^. Tregs serve the important role of dampening and halting immune responses to prevent autoimmunity or chronic inflammation and also have a role in the induction and maintenance of allograft tolerance in solid organ transplantation^2–4^. Until now, much of what is known about Tregs has been learned from CD4^+^FOXP3^+^ Treg. Much less is known about the CD8 counterpart, CD8^+^ Tregs. Accumulating evidence indicates that CD8^+^Tregs are also essential participants in normal and pathogenic immune responses^5–7^.

A role for CD8^+^ Tregs has also been suspected in autoimmune disease and allotransplantation^8^. The cells express many of the same cell surface molecules found on CD4^+^ Tregs. The thymus of healthy humans contains CD8^+^ T cells that express classical Treg markers (CD25, FOXP3, GITR, and CTLA-4) that exhibit immune suppressive effects through a contact-dependent mechanism^9^. CD8^+^CD25^+^FOXP3^+^ T cells influence self-reactive CD4^+^ T cells during the course of multiple sclerosis or colorectal cancer^10^. CD8^+^ T cells stimulated with a suboptimal dose of anti-CD3 antibodies in the presence of interleukin (IL)-15 express C-C chemokine receptor type 7 (CCR7) and acquire new functions and differentiate into immunosuppressive T cells^11^. The CCR7^+^CD8^+^ T cells avidly express FOXP3 and prevent CD4^+^ T cells from differentiating at a very early stage. The immune suppressive effect of CCR7^+^CD8^+^ T cells was supported by other results^12^.

The role of the CCR7^+^CD8^+^ T-cell phenotype has not been fully investigated in kidney transplants (KT), nor has its inhibitory role against alloreactive T cells, involved in the development of allograft rejection. To address these knowledge gaps, we formulated an induction protocol for CCR7^+^CD8^+^ T-cell expansion *in vitro*. Second, we investigated whether expanded CCR7^+^CD8^+^ T cells could regulate pathogenic effector T cells in an *in vitro* transplantation model using T-cell activation conditions or coculture system with human renal proximal tubular epithelial cells (HRPTEpiC). Lastly, we investigated the clinical significance of CCR7^+^CD8^+^ T cells in KT in an *ex vivo* analysis of peripheral blood mononuclear cells (PBMCs) isolated from KT recipients with or without T-cell mediated rejection (TCMR).

## Results

### Expansion of CCR7^+^CD8^+^ T cells with anti-CD3, IL-15, IL-2, and retinoic acid

**Figure 1:**
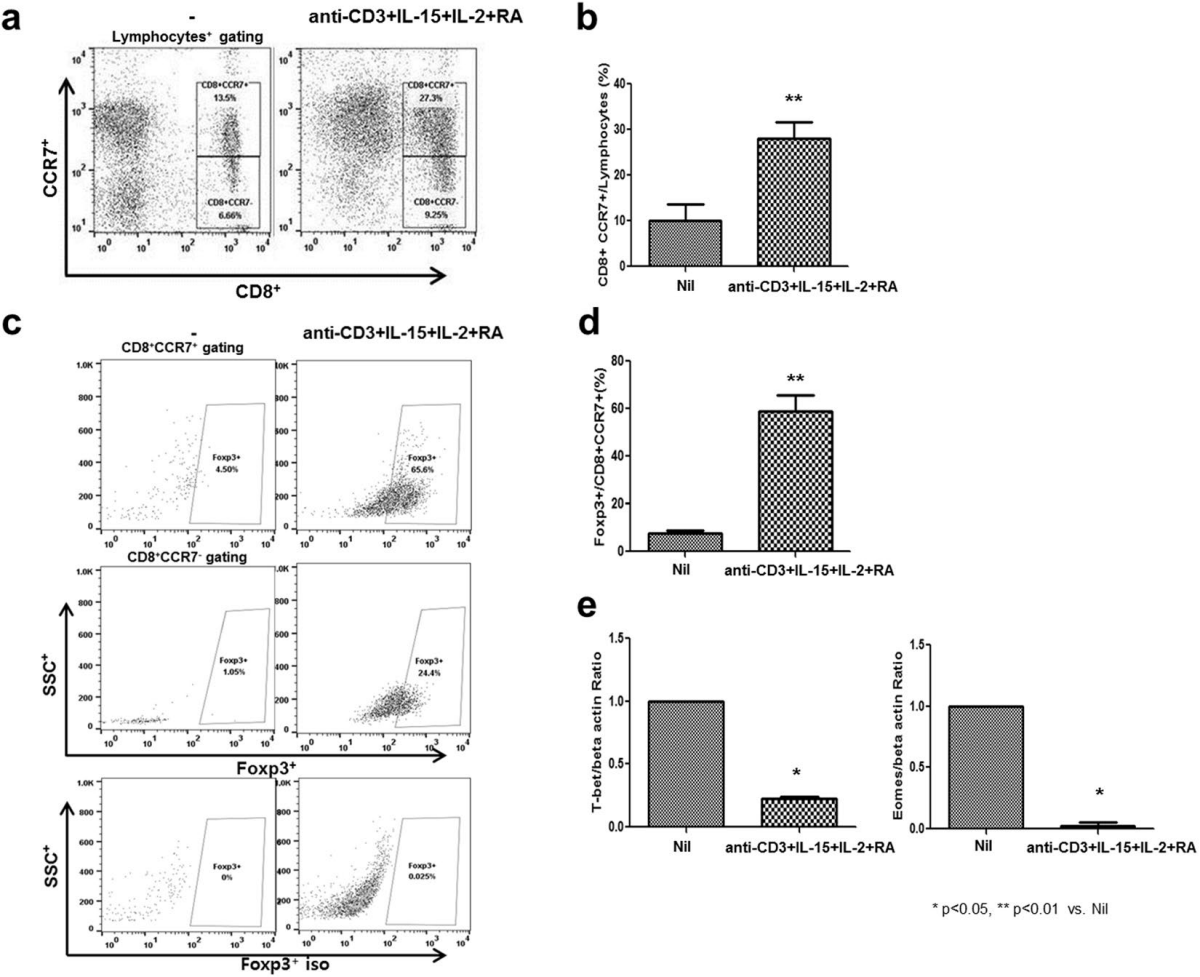
Induction and expansion of CCR7^+^CD8^+^ T cells. PBMCs (n = 5) were collected from healthy individuals, plated at 2 × 10^5^ cells per well and stimulated with anti-CD3 Abs (0.1 μg/ml), recombinant IL-15 (20 ng/ml), IL-2 (20 ng/ml), and retinoic acid (1 μg/ml). On day 3, cells were harvested, stained with antibodies specific to CD8, CCR7, and Foxp3, and analyzed by flow cytometry. The percentage of CCR7^+^ cells was determined using cells gated for CD8^+^ (**a**,**b**). The percentage of the Foxp3^+^ and Foxp3^+^ isotype was determined using cells gated for CD8^+^CCR7^+^ (**c**,**d**). **(e)** T-bet and Eomes mRNA expression was by real-time PCR. Bars represent the median with range. ^*^p < 0.05, ^**^p < 0.01 vs. Nil.

To determine the expansion protocol for CCR7^+^CD8^+^ T cells, isolated PBMCs were stimulated using anti-CD3, IL-15, IL-2, and retinoic acid. We included appropriate isotype controls in Fig. 1a,c. The protocol successfully stimulated the expansion of about 30% of CCR7^+^/CD8^+^ T cells from around 10% for the Nil condition (Fig. 1a,b) to about 50% of FOXP3^+^/CCR7^+^CD8^+^ T cells from around 5% for the nil condition (p < 0.05 vs. Nil for each) (Fig. 1,d). The CCR7^+^CD8^+^ induction protocol significantly reduced the expression of T-bet and Eomes in contrast with the concern that the level of these inflammatory markers may be raised using this induction protocol (Fig. 1e) (p < 0.05 vs. Nil). In addition, the CCR7^+^CD8^+^ induction protocol significantly increased the percentage of PD-1^+^/CD8^+^CCR7^+^, CD25^+^/CD8^+^CCR7^+^, Granzyme B^+^/CD8^+^CCR7^+^, and GITR^+^/CD8^+^CCR7^+^ T cells compared to the Nil (Supplementary Fig. S1)

### Suppressive effect of CCR7^+^CD8^+^ T cells on activated CD4^+^ T-cell

**Figure 2:**
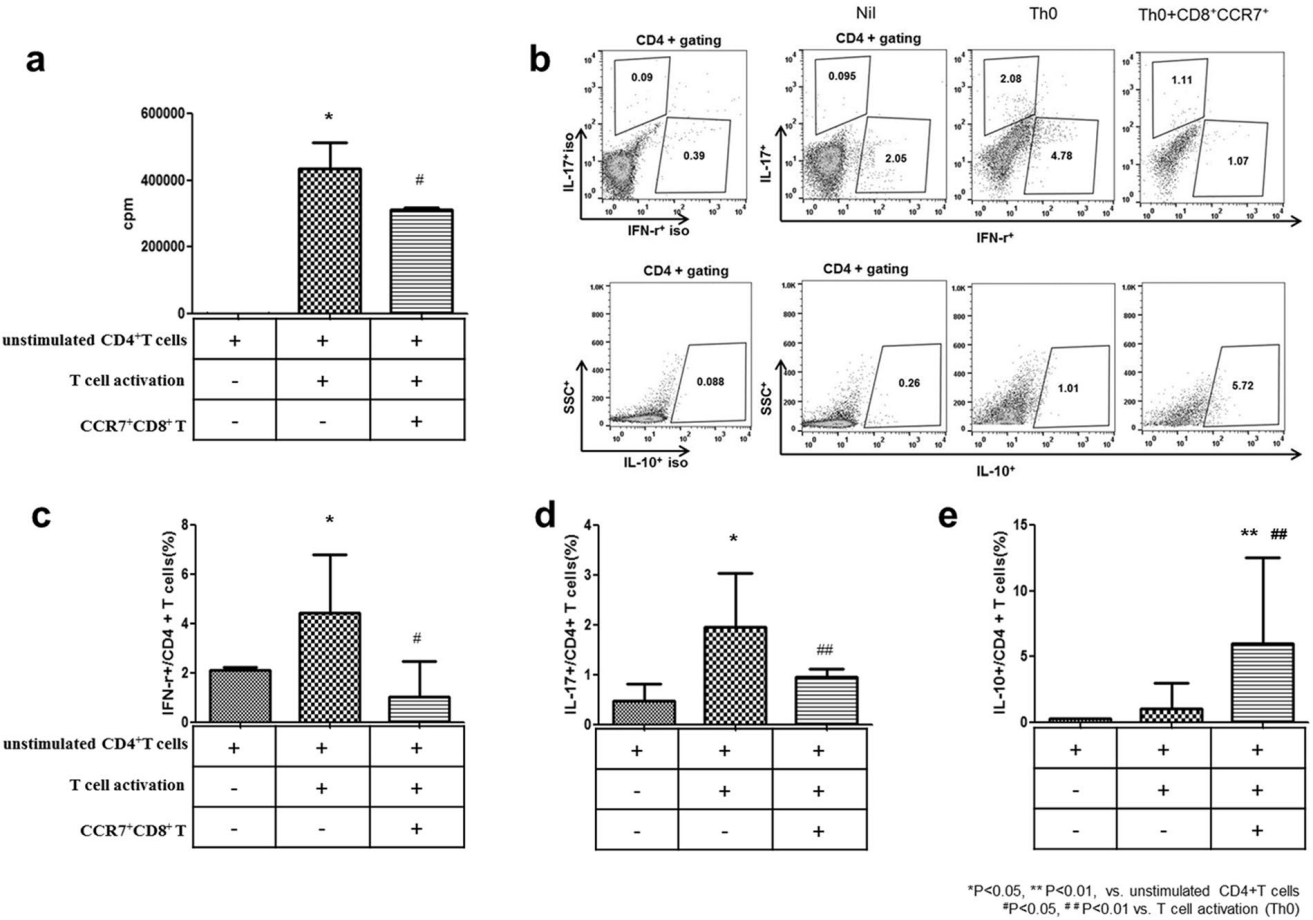
CCR7^+^CD8^+^ T cell-mediated suppression of activation of isolated CD4^+^ T cells from healthy donors. They were then cultured under CD4^+^ T-cell activation conditions with anti-CD3 and anti-CD28 for 72 hours (n = 6). (**a**) CCR7^+^CD8^+^ T cell-mediated suppression of the proliferation of T cells within the PBMC population isolated from healthy donors was measured using a ^3^H-thymidine incorporation assay. The cells were cultured for 3 days. (**b**) PBMCs were stained with PE-cy7-CD4, FITC-IFN-γ, PE-IL-17, and APC-IL-10. The proportion of (**c**) IFN-γ^+^ in the CD4^+^ T cells (%), (**d**) IL-17^+^ in the CD4^+^ T cells (%), and (**e**) IL-10^+^ in the CD4^+^ T cells (%) was performed using flow cytometry. ^*^p < 0.05, ^**^p < 0.01 vs. unstimulated CD4^+^ T cells, ^#^p < 0.05, ^##^p < 0.01 vs. T cell activation (Th0 condition).

Incubation of PBMCs isolated from healthy donors with CCR7^+^CD8^+^ T cells significantly suppressed the proliferation of CD4^+^ T cells compared to the Th0 condition (Fig. 2a). Next, the impact of CCR7^+^CD8^+^ T cells in CD4^+^ T cells was investigated. We included appropriate isotype controls in Fig. 2b. Incubation with CCR7^+^CD8^+^ T cells resulted in a significant decrease in the proportion of IFN-γ^+^/CD4^+^ T cells (1.0 ± 0.7% CCR7^+^CD8^+^ T cells vs. 4.4 ± 1.3% Th0 condition alone), and IL-17^+^/CD4^+^ T cells (0.9 ± 0.2% CCR7^+^CD8^+^ T cells vs. 1.9 ± 0.5% Th0 condition) (Fig. 2b–d). In contrast, an increase in IL-10^+^/CD4^+^ T cells (5.9 ± 2.7%) was detected in the CCR7^+^CD8^+^ T cells coculture condition, compared to the Th0 condition alone (1.0 ± 1.0%) or Nil (Fig. 2e) (^*^p < 0.05, ^**^p < 0.01 vs. unstimulated CD4+ T cells ^#^p < 0.05, ^# #^p < 0.01 vs. Th0 condition).

### Suppressive effect of CCR7^+^CD8^+^ T cells on inflammatory cytokine production from activated CD4^+^ T cells

**Figure 3:**
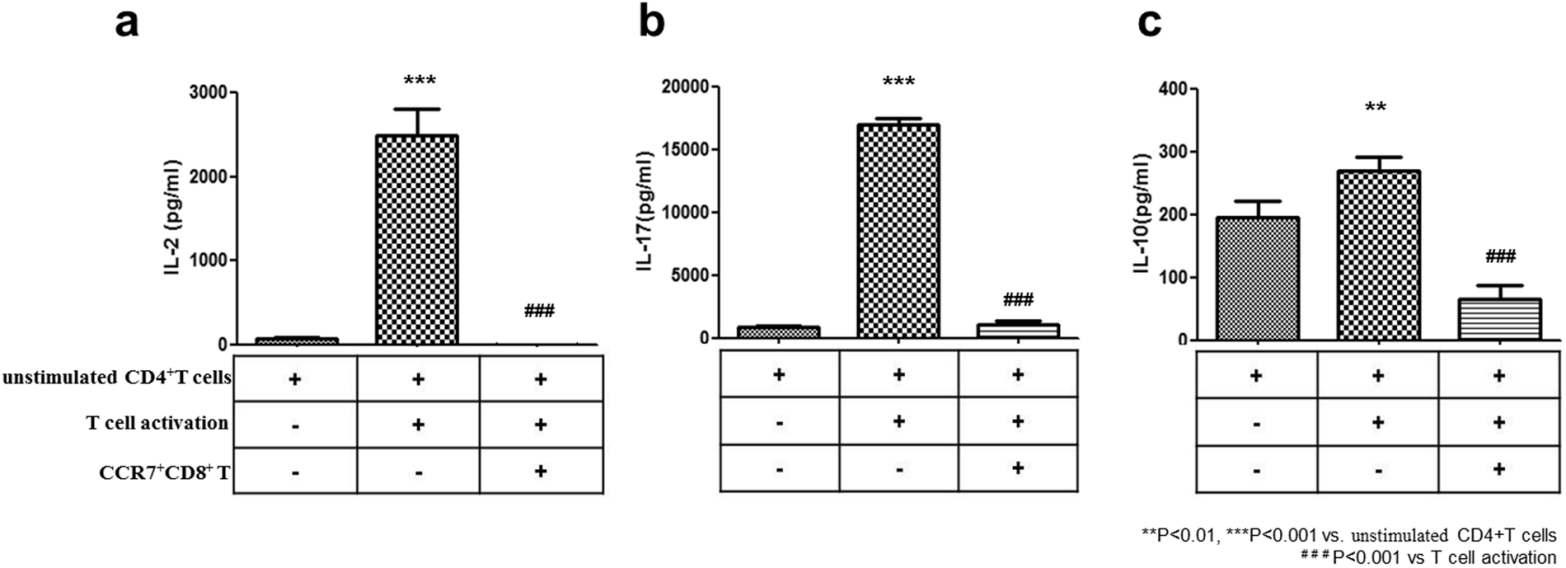
Effect of CCR7^+^CD8^+^ T cell-mediated suppression on activated CD4^+^ T-cell cytokine levels. To induce CD4^+^ T cell activation, CD4^+^ T cells (5 × 10^5^) (n = 4) were incubated for 72 h with anti-CD3 and anti-CD28. To investigate the suppressive effects of CCR7^+^CD8^+^ T cells, stimulated CD4^+^ T cells were cultured with CCR7^+^CD8^+^ T cells. The secretion of (**a**) IL-2, (**b**) IL-17, and (**c**) IL-10 by stimulated CD4^+^ T cells was performed by ELISA of the culture supernatant. Note that addition of CCR7^+^CD8^+^ T cells significantly decrease IL-2, IL-17, and IL-10 levels that were increased by stimulated CD4^+^ T cells. ^**^p < 0.01, ^***^p < 0.001 vs. unstimulated CD4^+^ T cells and ^###^*p* < 0.001 vs. stimulated CD4^+^T. Values are the median with range of triplicate cultures.

In the T-cell activation condition, the secretion level of IL-2 (2493 ± 222 pg/ml), IL-17 (17026 ± 581 pg/ml), and IL-10 (269 ± 24 pg/ml) were significantly increased in comparison with the Nil condition (IL-2, 79 ± 9 pg/ml; IL-17, 959 ± 139 pg/ml; and IL-10, 195 ± 28 pg/ml). Meanwhile, addition of CCR7^+^CD8^+^ T cells significantly suppressed IL-2 (6 ± 1 pg/ml) and IL-17 (1092 ± 336 pg/ml) protein levels compared to CD4^+^ T-cell activation alone (IL-2, 2493 ± 222 pg/ml, IL-17; 17026 ± 581 pg/ml) (Fig. 3a,b). Incubation with CCR7^+^CD8^+^ T cells did suppress IL-10 (66 ± 16 pg/ml) protein levels compared to CD4^+^ T-cell activation condition alone (269 ± 24 pg/ml) (Fig. 3c) (**p < 0.01, ***p < 0.001 vs. unstimulated CD4 + T cells, ^###^p < 0.001 vs Th0 condition).

### Suppressive effect of CCR7^+^CD8^+^ T cells on the differentiation of T cells into effector cells after cocultured with HRPTEpiC

**Figure 4:**
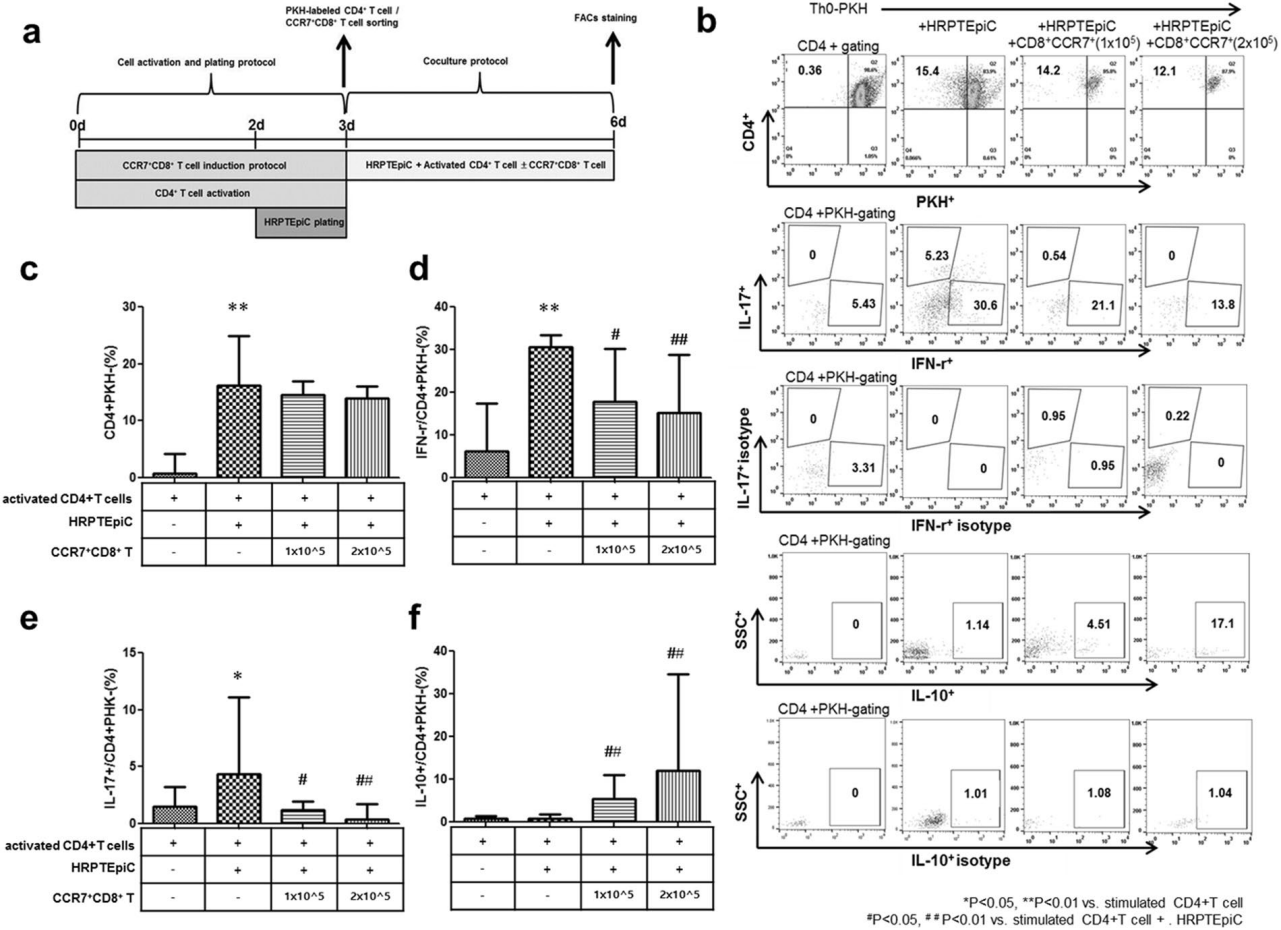
Effect of CCR7^+^CD8^+^ T cell-mediated suppression on T-cell proliferation by contact with HRPTEpiC. (**a**) The protocol for T-cell and HRPTEpiC coculturing. (**b**) The percentages of IFN-γ, IL-17, and IL-10^+^ in the CD4^+^T PKH^−^ cells were obtained by flow cytometry. Activated CD4^+^ T cells were cultured with HRPTEpiC for 72 h, and the percentage of (**c**) CD4^+^PKH^−^, (**d**) IFN-γ^+^/CD4^+^PKH^−^, (**e**) IL-17^+^/CD4^+^PKH^−^, and (**f**) IL-10^+^/CD4^+^PKH^−^ was performed by flow cytometry (n = 6). Note that addition of CCR7^+^CD8^+^ T cells significantly decreased CD4^+^PKH^−^, IFN-γ^+^/CD4^+^PKH^−^, and IL-17^+^/CD4^+^PKH^−^ levels, which was further increased with HRPTEpiC. ^*^p < 0.05, ^**^p < 0.01 vs. stimulated CD4^+^ T cells and ^#^p* < *0.05, ^##^p < 0.01 vs. stimulated CD4^+^ T cells + HRPTEpiC. Values are the median with range of triplicate cultures.

Figure 4a showed the coculture system of HRPTEpiC and stimulated T cells (see method for detail). We included appropriate isotype controls in Fig. 4b. Coculture of HRPTEpiC with stimulated T cells under the CD4^+^ T-cell activation condition significantly increased T-cell proliferation (PKH^−^) compared to without coculture condition (Fig. 4b). However, treatment with CCR7^+^CD8^+^ T cells significantly decreased the proliferation of CD4^+^ T cells (CD4^+^PKH^−^) compared to stimulated CD4^+^ T-cell cocultured with HRPTEpiC (Fig. 4c). When we investigated the effect of CCR7^+^CD8^+^ T cells on the differentiation into CD4^+^ T-cell subtype, treatment with CCR7^+^CD8^+^ T cells significantly suppressed the proportion of IFN-γ^+^/CD4^+^PKH^−^cells (17.9 ± 8.0% [+CCR7^+^CD8^+^ T cells (1 × 10^5^)] and 15.2 ± 6.9% [+CCR7^+^CD8^+^ T cells (2 × 10^5^)]) and also IL-17^+^/CD4^+^PKH^−^cells (1.2 ± 0.6% [+CCR7^+^CD8^+^ T cells (1 × 10^5^)] and 0.3 ± 0.6% [+CCR7^+^CD8^+^ T cells (2 × 10^5^)]) compared to stimulated CD4^+^ T cells cocultured with HRPTEpiC (IFN-γ^+^/CD4^+^ PKH^−^cells; 30.5 ± 5.0%), (IL-17^+^/CD4^+^PKH^−^cells 4.3 ± 2.7%) (Fig. 4d,e). However, treatment with CCR7^+^CD8^+^ T cells significantly increased the proportion of IL-10^+^/CD4^+^PKH^−^ cells (5.4 ± 2.7% [+CCR7^+^CD8^+^ T cells (1 × 10^5^)] and 12.1 ± 8.3% [+CCR7^+^CD8^+^ T cells (2 × 10^5^)]) compared to stimulated CD4^+^ T cells cocultured with HRPTEpiC (0.9 ± 0.4%) (Fig. 4f) (^*^p < 0.05, ^**^p < 0.01 vs. stimulated CD4+ T cell ^#^p < 0.05, ^##^ p < 0.01 vs. stimulated CD4+ T cell+ HRPTEpiC).

### Suppressive effect of CCR7^+^CD8^+^ T cells on inflammatory cytokine production from effector T cells after coculture with HRPTEpiC

**Figure 5:**
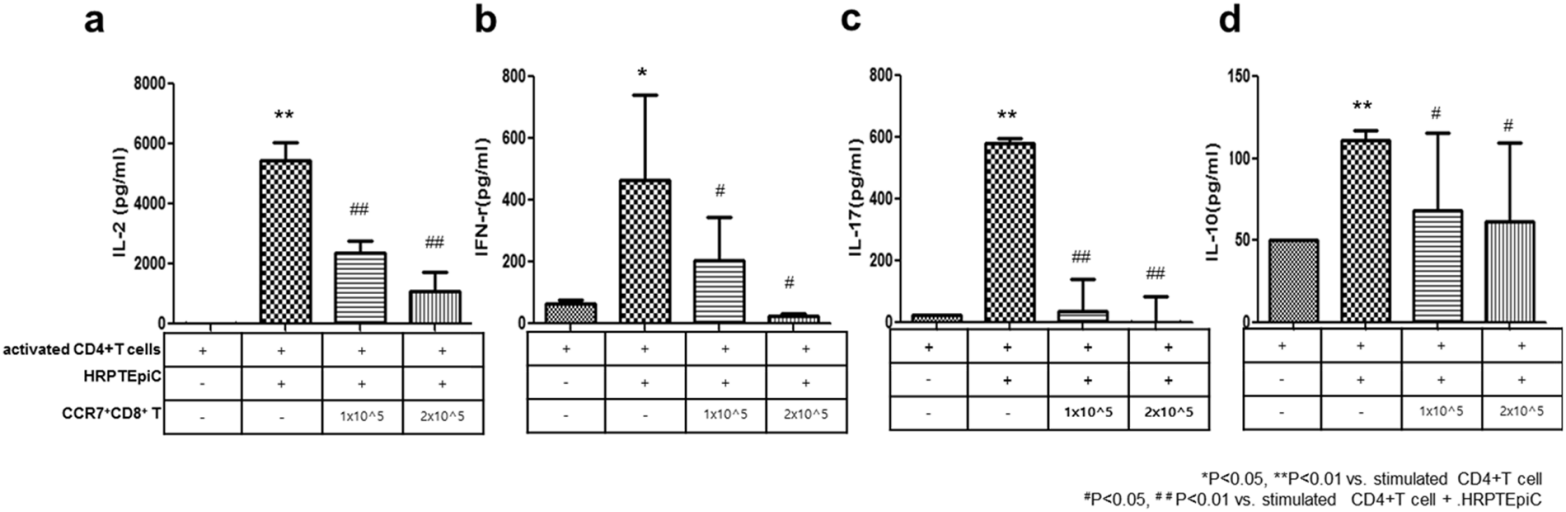
Effect of CCR7^+^CD8^+^ T cell-mediated suppression on inflammatory cytokines production by stimulated CD4^+^ T cells cocultured with HRPTEpiC. (**a**) IL-2, (**b**) IFN-γ, (**c**) IL-17, and (**d**) IL-10 secretion by stimulated CD4^+^ T cells cocultured with/without CCR7^+^CD8^+^ T cells (as indicated) cocultured for 72 hours with HRPTEpiC (n = 6). Note that CCR7^+^CD8^+^ T cells suppressed the CD4^+^ T-cell production of IL-2, IFN-γ, and IL-17. ^*^p < 0.05, ^**^p < 0.01 vs. stimulated CD4^+^ T cells and ^#^p* < *0.05, ^##^p < 0.01 vs. stimulated CD4^+^ T-cell + HRPTEpiC. Values are expressed as the median with range of triplicate cultures.

Coculture of HRPTEpiC with stimulated CD4^+^ T cells meaningfully increased the secretion of IL-2 (5431 ± 484 pg/ml [+stimulated CD4^+^ T cells+ HRPTEpiC]), IFN-γ (466 ± 198 pg/ml [+stimulated CD4^+^ T cells+ HRPTEpiC]), IL-17 (580 ± 22 pg/ml [+stimulated CD4^+^ T cells+ HRPTEpiC]) secretion from T cells compared to HRPTEpiC alone condition (16 ± 0 pg/ml [IL-2], 64 ± 10 pg/ml [IFN-γ], 25 ± 0 pg/ml [IL-17]). However, addition of CCR7^+^CD8^+^ T cells suppressed the secretion of those inflammatory cytokines compared to stimulated CD4^+^ T cells cocultured with HRPTEpiC (Fig. 5a–c). However, addition of CCR7^+^CD8^+^ T cells did not decrease the secretion of IL-10 compared to stimulated CD4^+^ T cells cocultured with HRPTEpiC (Fig. 5d) (^*^p < 0.05, ^**^p < 0.01 vs. stimulated CD4 + T cell, ^#^p < 0.05, ^##^p < 0.01 vs. stimulated CD4+ T cell+ HRPTEpiC).

### Proportion of CCR7^+^CD8^+^ T cells and effector T-cell subsets in peripheral blood from kidney transplant recipients with or without TCMR

**Figure 6:**
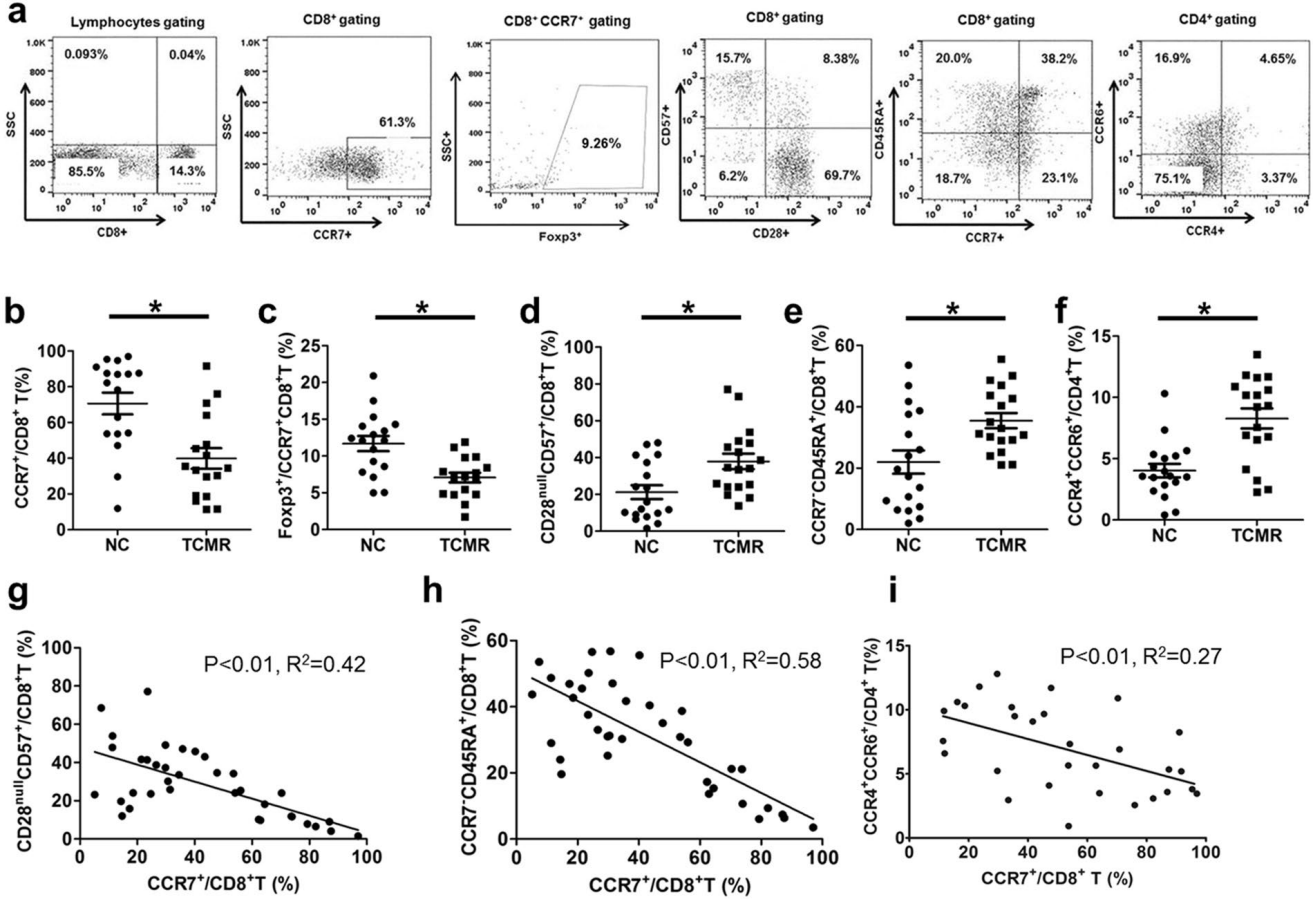
Comparison of CCR7^+^CD8^+^ T and effector T cells in PBMCs from kidney transplant recipients with or without TCMR. (**a**) The representative figure for the flow cytometric analysis of CCR7^+^ CD8^+^, Foxp3^+^/CCR7^+^CD8^+^, CD28^null^CD57^+^, CD45RA^+^CCR7^−^/CD8^+^, and CCR4^+^CCR6^+^/CD4^+^ T cells. Panels (b–f) show the comparison of the distribution of (**b**) CCR7^+^/CD8^+^ (**c**) Foxp3^+^/CCR7^+^CD8^+^, (**d**) CD28^null^CD57^+^/CD8^+^, (**e**) CD45RA^+^CCR7^−^/CD8^+^ T (CD8^+^TEMRA), and (**f**) CCR4^+^CCR6^+^/CD4^+^ T within the peripheral blood mononuclear cells (PBMCs) between the NC (n = 17) and TCMR group (n = 17). (**g**–**i**) shows the correlation curve between CCR7^+^/CD8^+^ T with (**g**) CD28^null^CD57^+^/CD8^+^, (**h**) CD45RA^+^CCR7^−^/CD8^+^, and (**i**) CCR4^+^CCR6^+^/CD4^+^ T cells. ^*^p < 0.05 between NC and TCMR.

Figure 6a shows representative flow cytometric data for CCR7^+^/CD8^+^, CD57^+^CD28^null^/CD8^+^, CCR7^−^CD45RA^+^/CD8^+^, and CCR4^+^CCR6^+^/CD4^+^ T cells. The percentage of CCR7^+^/CD8^+^ T cells was significantly decreased in the TCMR group in comparison with the NC group (p < 0.01). The proportion of FoxP3^+^/CCR7^+^CD8^+^ T cells was also significantly decreased in the TCMR group in comparison with the NC group (p < 0.05). In contrast, the proportion of CD57^+^CD28^null^/CD8^+^ and CCR4^+^CCR6^+^/CD4^+^ T cells was significantly higher than the NC group (p < 0.05) and CCR7^−^CD45RA^+^/CD8^+^ T cells showed a higher tendency (p = 0.06) in the TCMR group in comparison with those in the NC group. (Fig. 6b–f) In addition, the proportion of CCR7^+^/CD8^+^ T cells showed a significant negative correlation with CD57^+^CD28^null^/CD8^+^ (p < 0.01, R^2^ = 0.76), CCR7^−^CD45RA^+^/CD8^+^ (p < 0.01, R^2^ = 0.61), and CCR4^+^CCR6^+^/CD4^+^ T cells (p < 0.01, R^2^ = 0.27) (Fig. 6g–i).

## Discussion

In this study, the *in vitro* suppressive effect of CCR7^+^CD8^+^ T cells was investigated using conditions that mimicked allograft rejection and also investigated whether CCR7^+^CD8^+^ T cells are clinically significant in an *ex vivo* study using PBMCs from KT recipients with or without TCMR. CCR7^+^CD8^+^ T cells effectively suppressed T-cell proliferation or differentiation in two kinds of *in vitro* transplant models. In addition, the proportion of CCR7^+^/CD8^+^ T cells in PBMCs negatively correlated with three types of effector T cells. All of these findings suggest that CCR7^+^CD8^+^ T cells may have a role in the suppression of effector T cells in KT recipients.

For this research, the appropriate conditions for the expansion of CCR7^+^CD8^+^ T cells with a regulatory function were determined because each Treg population has unique conditions for induction and the mechanisms through which it functions^13^. The regulatory function of CCR7^+^CD8^+^ T cells can be induced by the combined signal of T-cell receptor (TCR)-crosslinking anti-CD3 antibodies and IL-15-mediated STAT5 signaling^14^. STAT5 has been implicated in regulating FOXP3 transcription^15^. In addition, all*-trans* retinoic acid has a completely differential role in promoting the phenotypic and functional development of TGF-β-induced CD4^+^ and CD8^+^ Foxp3^+^ Tregs^16^. Finally, the induction protocol using anti-CD3, IL-15, IL-2, and retinoic acid successfully induced the expression of FOXP3-related regulatory markers in CCR7^+^CD8^+^ T cells and reduced the expression of inflammatory markers such as Eomes and T-bet.

Next, two separate *in vitro* studies using T cells isolated from PBMCs and the HRPTEpiC line were designed. First, in an *in vitro* study using PBMCs, a well-established *in vitro* model utilizing a T-cell activation condition was used. CD4^+^ T cells were selected as target effector T cells, where if CD8^+^ T cells were used as the target, it would be difficult to assess whether the proliferating cells were target cells or CCR7^+^CD8^+^ T cells^17,18^. Coculturing of CCR7^+^CD8^+^ T cells with stimulated CD4^+^ T cells meaningfully decreased the proliferation of CD4^+^ T cells under activation conditions in a mixed lymphocyte reaction. In addition, it significantly decreased differentiation into IFN-γ- or IL-17^−^ positive CD4^+^ T cells and decreased the level of IL-2 and IL-17 in the culture supernatant. The results are compatible with a previous study that showed the suppressive effect of CCR7^+^CD8^+^ T cells on the proliferation and differentiation of T cells^19^. In contrast, IL-10-positive CD4^+^ T cells and the IL-10 level increased in the CCR7^+^CD8^+^ T-cell treatment condition. Thus, CCR7^+^CD8^+^ T cells may facilitate the induction of regulatory CD4^+^ T cells and regulatory cytokines that further suppress other effector T cells, as reported previously^11,20,21^.

The differentiation pattern of T cells from kidney allografts during TCMR may differ from that observed with cytokine stimulation^22^. Therefore, in this study, HRPTEpiC and a T-cell coculture system were used as presented in other previous reports^23,24^. HRPTEpiCs are not only the main target of alloreactive T cells but are also important T-cell modulators^25^. Various molecules involved in the transmission of immune cell regulation signals are expressed or can be induced on the surface of HRPTEpiCs, and may directly affect the proliferation of T cells by a contact mechanism^26,27^. All of these mechanisms may induce characteristic T-cell differentiation patterns during rejection. In this study, the proportion of PKH-negative T cells (the proliferating portion) significantly increased after coculturing, as expected. In PKH-negative CD4^+^ T-cell gating, the proportion of IFN-γ- and IL-17-positive cells increased. It is possible that activated T cells activate HRPTEpiC to express adhesion molecules or to secrete inflammatory cytokines or chemokines^23,28^. These HRPTEpiC reactions may induce T-cell proliferation or differentiation into effector CD4^+^ T cells.

In the presence of CCR7^+^CD8^+^ T cells, the proportion of PKH-negative T cells significantly decreased. A significant decrease was also apparent for the differentiation into CD4^+^ T cells that were positive for IFN-γ and IL-17, and IL-2, IFN-γ, and IL-17 levels in the culture supernatant. The results suggest that CCR7^+^CD8^+^ T cells effectively suppress the proliferation and/or differentiation of CD4^+^ T cells after contact with HRPTEpiC. In contrast, the proliferation of IL-10-positive cells significantly increased after coculturing with CCR7^+^CD8^+^ T cells. In addition, IL-10 levels were less significantly affected by the addition of CCR7^+^CD8^+^ T cells in comparison with other inflammatory cytokines.

In regard to IL-10 in both *in vitro* studies, IL-10^+^ intracellular staining in CD4^+^ T cells and IL-10 level in the culture supernatant did not synchronize with each other. The first possible explanation for this finding is that IL-10 levels may reflect not only production from T cells but also from other cell types. Indeed, IL-10 is known to be produced by a variety of innate and adaptive immune cells, including macrophages, dendritic cells (DCs), natural killer (NK) cells, CD4, CD8, γδ T cells, and B cells^29,30^. The effect of CCR7^+^CD8^+^ T cells on IL-10 secretion from these cell types can be different from in the effect of CD4^+^ T cells and it may result in discordant results between serum IL-10 levels and IL-10 positive CD4^+^ T cells. Second, it may be because of measurement differences between flow cytometry and the enzyme-linked immunosorbent assay (ELISA). Using flow cytometry, the expression of IL-10 in CD4^+^ T cells can be assessed at the time of the measurement. In contrast, IL-10 levels may represent an accumulating amount of IL-10 during the 72 h incubation time. Therefore, it is possible that the IL-10 level may not differ significantly between nil and CCR7^+^CD8^+^ T-cell treatment conditions in contrast to flowcytometry.

The effector CD4^+^ T-cell regulation mechanism of CCR7^+^CD8^+^ T cells is unclear, but it is well known that regulatory T cells may use the perforin-granzyme pathway as a mechanism to suppress the function of immune cells by killing them^31^. In addition, IFN-γ has an indispensable role in the suppressive effect of regulatory T cells in the prevention of autoimmune disease^32^. Therefore, IFN-γ, perforin, and granzyme B staining were compared between the nil and CCR7^+^CD8^+^ induction (anti-CD3^+^IL-15^+^ IL-2^+^RA) condition. Finally, the proportion of IFN-γ-, perforin-, and granzyme B-positive cells among the CCR7^+^CD8^+^ T cells was significantly increased in the induction condition (Supplementary Fig. S2). Therefore, the increased expression of members of the perforin-granzyme pathway and also IFN-γ observed using the CCR7^+^CD8^+^ T induction protocol may involve the regulatory function of CCR7^+^CD8^+^ T cells.

Lastly, the significance of CCR7^+^CD8^+^ T cells in KT recipients was assessed by *ex vivo* analysis of isolated PBMCs. Along with the proportion of CCR7^+^/CD8^+^ T cells, three effector T cells were analyzed: CD57^+^CD28^null^/CD8^+^ immune senescent, CCR7^-^CD45RA^+^/CD8^+^ (TEMRA), and CCR4^+^CCR6^+^/CD4^+^ T cells, which are involved in the development of acute rejection^18,33–35^. Both CD57^+^CD28^null^CD8^+^ and CCR7^-^CD45RA^+^CD8^+^ T cells belong to the end-differentiated effector cell state, whose features are contradictory to that of CCR7^+^CD8^+^ T cells, which display a naïve cell state^24,36^. In addition, expression of CCR4^+^ and CCR6^+^ bestows the capacity to migrate to the site of inflammation^37,38^. CCR7^+^/CD8^+^ T cells and also the FOXP3 expression in this cell type were decreased in the TCMR group in comparison with the NC group, while all three effector T-cell types were significantly increased in the TCMR group. In addition, a significant negative correlation was identified between CCR7^+^/CD8^+^ T cells and all three types of effector T cells, suggestive of the suppressive effects of CCR7+ CD8+ T cells on other effector T cells.

The proportion of conventional CD4^+^ Treg (CD127^low^CD25^high^/CD4^+^ T) in the same patient groups was also analyzed. In contrast to CCR7^+^CD8^+^ T cells, which seek out and interfere with the immune response of the T-cell zones of secondary lymphoid organs rather than in the target organ^39,40^, CD4^+^ Treg rapidly invaded the target organ and showed a suppressive effect via a paracrine function or by direct contact with effector T cells^41^. Previous research on circulating CD4^+^ Tregs did not show positive results, in contrast with the promising results from *in vitro* studies on Treg infiltration in allograft tissue^42–44^. In the present study, the percentage of CD127^low^CD25^high^/CD4^+^ T cells was similar between the normal biopsy and TCMR groups, with no significant correlation to any of the three effector T cells evident (Supplementary Fig. S3). This suggests that CCR7^+^CD8^+^ T cells are more useful than CD4^+^ Tregs for monitoring the clinical status of KTRs using peripheral blood.

Our study has some limitations. First, the effect of CCR7^+^CD8^+^ T cells on the interaction between tubule cells and T cells does not account for all facets of allograft rejection. Other studies using vascular endothelial cells and B cells that are also involved in alloimmunity may be necessary to reflect the full spectrum of allograft rejection. Second, in the *ex vivo* study, there was a correlation between CD8^+^/CCR7^+^ T cells and the proportion of effector T cells in the peripheral blood. This did not directly prove the immune suppressive effect of CD8^+^CCR7^+^ T cells on effector T cells. Third, there are concerns regarding the phenotype and effector function profile of CCR7+ CD8+ T cells in the *ex vivo* study. It is true that not all CCR7^+^CD8^+^ T cells are regulatory T cells. However, CCR7^+^CD8^+^ T cells can show immune suppressive effects according to the circumstances. Indeed, FOXP3 expression in CCR7^+^CD8^+^ T cells was significantly higher than in the TCMR group, but IL-17 expression was significantly lower in the NC group in comparison with the TCMR group (Supplementary Fig. S4). These findings suggest that a higher proportion of CCR7^+^CD8^+^ T cells in the normal biopsy control group can be a marker of the immune suppressed state rather than pathogenic status. Lastly, it can be necessary to investigate the infiltration of CCR7^+^CD8^+^ T cells in the allograft tissue because circulating cell populations can be different to those found in tissues. However, further investigation may be required for clarification.

In conclusion, CCR7^+^CD8^+^ T cells regulate the proliferation and/or differentiation of T cells into effector cells under T-cell activating conditions and during coculture with HRPTEpiC. In addition, there was a significant negative correlation between CCR7^+^CD8^+^ T cells and various types of effector T cells in an *ex vivo* study, suggesting that some interaction between the cell types is involved in the development or suppression of TCMR. The results suggest that the use of CCR7^+^CD8^+^ T cells may be considered as an important immune suppression strategy for KT recipients.

## Methods

### Ethics statement, patient populations, and study design

Two separate *in vitro* experiments and an *ex vivo* studies were designed. First, the suppressive effect of CCR7^+^CD8^+^ T cells on the activated CD4^+^ T cells was evaluated using PBMCs. Six healthy individuals aged 27–40 years were recruited for blood donation. The mixed lymphocyte reaction was used for the proliferation of CD4^+^ T cells with CCR7^+^CD8^+^ T cells, and flow cytometry was used for the differentiation of unstimulated CD4^+^ T cells into IFN-γ, IL-17, or IL-10 positive T cells under T-cell activating conditions. Cytokine levels (IL-2, IL-17, IL-10) were also assessed by ELISA. Second, the suppressive effect of CCR7^+^CD8^+^ T cells on the proliferation of CD4^+^ T cells into IFN-γ, IL-17, or IL-10 positive T cells was investigated using PKH-69-labeled T cells by flow cytometry in the coculture system with HRPTEpiC. T-cell specific cytokine levels (IL-2, IFN-γ, IL-17, and IL-10) in the culture supernatant were also measured.

An *ex vivo* study was performed to investigate the proportion of CCR7^+^CD8^+^ T cells using PBMCs isolated from 34 KT recipients at the time of allograft biopsy. Seventeen recipients with normal biopsy findings without any evidence of rejection comprised the normal control group (NC). Another 17 patients who showed T cell-mediated rejection (TCMR) according to the 2007 Banff classification comprised the TCMR group^45^. All patients were taking tacrolimus and mycophenolate mofetil combination therapy. The baseline characteristics of both groups are presented in Table 1. Flow cytometry was used to analyze the proportions of CCR7^+^CD8^+^ and effector T cells, such as immune senescent T cells (CD57^+^CD28^null^CD8^+^ T) or TEMRA (CCR7^-^CD45RA^+^CD8^+^) and CCR4^+^CCR6^+^CD4^+^ T cells. All methods were performed in accordance with the relevant guidelines and regulations. Written informed consent was obtained from KT recipients and healthy individuals. The protocol for this study was approved by the Institutional Review Board of Seoul St. Mary’s Hospital (KC13TNMI0701).

**Table 1:** Baseline characteristics of the kidney transplant recipients included in *ex vivo* study.

	**Normal biopsy** **(n = 17)**	**TCMR** **(n = 17)**	***P***
Age (year)	45.9 ± 10.1	44.9 ± 10.0	0.77
Male, n (%)	13 (77)	13 (77)	1.0
Post-transplant month	2.9 ± 1.4	3.6 ± 2.7	0.28
MDRD eGFR (mL/min/1.73 m^**2**^**)**	74.6 ± 36.3	43.0 ± 12.5	0.02
HLA mismatch number	4.1 ± 1.4	3.6 ± 1.7	0.28
Donor type			
Living donor, n (%)	10 (77)	11 (48)	0.73
Deceased donor, n (%)	7 (23)	6 (52)	
Induction therapy			
ATG, n (%)	3 (18)	3 (18)	1.00
Basiliximab, n (%)	14 (82)	14 (82)	
Maintenance immune suppression			
Tacrolimus dose	5.4 ± 0.4	5.2 ± 0.2	0.81
Trough level of tacrolimus	5.9 ± 0.6	6.1 ± 0.6	0.86
Steroid dose (5/10 mg), n (%)	13/4 (76/24)	13/4 (76/24)	1.00

TCMR, T-cell mediated rejection; MDRD, Modification of diet in renal disease; eGFR, estimated glomerular filtration rate; ATG, anti-thymocyte globulin; Tac, tacrolimus; CsA, cyclosporine.

### Cell isolation and culture

We collected peripheral blood for the analysis of immune cell profile and processed as follows. Peripheral blood mononuclear cells (PBMC) were isolated from blood by Ficoll–Hypaque (GE Healthcare, PA). PBMCs were cultured in RPMI medium as described previously^17,18^.

### CCR7^+^CD8^+^ T-cell induction protocol

PBMCs (1 × 10^6^) were stimulated with an anti-CD3 antibody with IL-15, IL-2, and retinoic acid. After incubation for six days, CD8^+^ T cells were purified by negative selection with the CD8^+^ T Cell Isolation Kit II (Miltenyi Biotec) followed by separation of CD8^+^CCR7^+^ T cells using PE-CCR7 antibody (BD Biosciences) and anti-PE microbeads (Miltenyi Biotec). The cells were then sorted using a FACS Aria device (Becton, Dickinson) or a MoFlo cell sorter (Beckman Coulter) to isolate CCR7^+^CD8^+^ cells. The cells were incubated with a monoclonal antibody against Foxp3 (fluorescein isothiocyanate [FITC], PCH101, IgG2a, κ; eBioscience), PD-1 (fluorescein isothiocyanate [FITC], J43, IgG1, κ; eBioscience), CD25 (APC, CD25-4E3, IgG2b, κ; eBioscience), Granzyme B (FITC, GB11, IgG1; eBioscience), GITR (APC, CD25-4E3, IgG2b, κ; eBioscience), or Granzyme B (FITC, 621, IgG1, κ; Biolegend). Isotype controls were monitored non-specific binding. The expression of FOXP3 in sorted CD8^+^CCR7^+^ T cells was compared across the different protocols.

### Real-time PCR

A LightCycler (Roche Diagnostics) was performed for PCR amplification. All the PCR reactions were performed according to the manufacturer’s instructions. The following primers for each molecule were used: *T-bet* sense, 5′-ACC AGC ATC AAA ATC CCA AG-3′; *T-bet* antisense, 5′- TTT CCA CAC TGC ACC CAC TT-3′; *Eomes* sense, 5′- AGC AAC CTG GGA CCA ACA AA-3′; *Eomes* antisense, 5′-GCC ATT GCA GGA AAG GTT GG-3′; *beta-actin* sense, 5′-GGA CTT CGA GCA AGA GAT GG-3′; and *beta-actin* antisense, 5′-TGT GTT GGC GAT CAG GTC TTT- G-3′. Melting curve analysis was performed immediately after the amplification protocol, under the following conditions: 0 seconds (hold time) at 95 °C, 15 seconds at 71 °C, and 0 seconds (hold time) at 95 °C. The temperature change rate was 20 °C/s except in the final step, during which it was 0.1 °C/s. The generated melting peak represented the quantity of the specific amplified product. The crossing point was defined as the maximum of the second derivative from the fluorescence curve. Negative controls that contained all elements of the reaction mixture except from the template DNA were also included. All samples were processed in duplicate.

### Suppression of CD4^+^ T-cell proliferation under T-cell stimulation by CCR7^+^CD8^+^ T cells

PBMCs were collected from six healthy donors and CD4^+^ T cells were isolated using monoclonal anti-human CD4 antibody conjugated to microbeads (Miltenyi Biotech). In the T-cell activation condition, PBMCs (5 × 10^5^) were incubated for 72 h with anti-CD3 antibody (1 μg/ml) and anti-CD28 antibody (1 μg/ml; 34019, R&D Systems, Inc.) before CD4^+^ T-cell isolation. CCR7^+^CD8^+^ T cells were isolated from the six donors as described above. For the suppression of CD4^+^ T cells by CCR7^+^CD8^+^ T cells, isolated CD4^+^ T cells (1 × 10^5^) were cocultured with T cell-depleted, irradiated APCs (1 × 10^5^) in the presence or absence of CCR7^+^CD8^+^ T cells (1 × 10^5^) for 3 days. The proliferation of CD4^+^ T cells was examined by adding ^3^H-thymidine (1 μCi/well; GE Healthcare) followed by incubation for 8 h. The level of ^3^H-thymidine incorporation was measured using a liquid β-scintillation counter (Beckman).

### Suppressive effects of CCR7^+^CD8^+^ T cells on the differentiation of CD4^+^ T cells under T-cell stimulation condition

Isolated CD4^+^ T cells from six healthy participants were incubated under various conditions for 72 h. To induce CD4^+^ T-cell activation, CD4^+^ T cells (5 × 10^5^) were incubated for 72 h with anti-CD3 antibody (1 μg/ml) and anti-CD28 antibody (1 μg/ml; 34019, R&D Systems, Inc.). To investigate the suppressive effects of CCR7^+^CD8^+^ T cells, CD4^+^ T cells were cultured with CCR7^+^CD8^+^ T cells. The cells were stained with PE/Cy7-CD4 monoclonal antibody. For staining intracellularly, the cells were incubated with monoclonal antibodies against IL-17, IFN-γ, and IL-10. Isotype controls were monitored non-specific binding. Cells were measured using a FACS Calibur flow cytometer and FlowJo software.

### Human renal proximal tubular epithelial cell (HRPTEpiC)

The HRPTEpiC line was purchased from ScienCell Research Laboratories. At first, CCR7^+^CD8^+^ T cells were expanded at day 0 as described above. PBMCs (1.5 × 10^5^) were incubated under T-cell activation conditions at day 0 for 72 h. On day 1, HRPTEpiC were seeded in plates. On day 3, isolated and PKH-labeled PBMC cells (1.5 × 10^5^ cells/well) were added with or without expanded and isolated CCR7^+^CD8^+^ T cells (1 × 10^5^ cells or 2 × 10^5^ cells/well). On day 6, the harvested cells were examined for proliferation using a FACSCalibur flow cytometer (BD Biosciences). All cultures were set up in triplicate (Fig. 4a).

### PKH-based assessment of T-cell proliferation after coculture with HRPTEpiC

PKH 67 (Sigma-Aldrich) was diluted according to the manufacturer’s directions. Briefly, 1.5 × 10^5^ PBMCs were washed in Dulbecco’s phosphate buffered saline (Gibco/BRL) and re-suspended in 1 ml of solution C from the kit. PKH 67 was diluted to 4 × 10^−6^ M in 1 ml of solution C. Cells were combined with dye and the tube was inverted several times over 3 mins. About 2 ml of FCS (BioWhittaker) was added to the tube and inverted continuously for 1 min. Cells were then transferred to a 15-ml conical tube with 4 ml of RPMI 1640 without Phenol Red (Sigma-Aldrich) with 10% FCS (BioWhittaker, Walkersville, MD) and washed three times in the same medium. All reagents and buffers were used at room temperature.

### ELISA

Cytokine production of IL-17, IL-2, IL-10, and IFN-γ in the culture supernatants from peripheral blood mononuclear cells or HRPTEpiC were performed using sandwich ELISA according to the manufacturer’s instructions.

### Flow cytometry

In the samples used for *in vitro* experiments, flow cytometry analysis was performed after collection of PBMCs. In the *in vitro* study, the cells were stained with monoclonal antibodies: PE/Cy7-CD4, APC-CD8 and APC-CD25. Staining for chemokine receptors was performed using anti-CCR7 (3D12, IgG2a, κ) mouse mAbs. For intracellular staining, the cells were incubated with monoclonal antibodies against PE-IL-17, FITC-IFN-γ, APC-IL-10, and FITC-Foxp3. Isotype controls were monitored non-specific binding. Cells were measured using a FACSCalibur flow cytometer and FlowJo software.

### *In vitro* reagents

Recombinant human IL-15 and IL-2 (R&D Systems) were purchased. Anti-CD3 and anti-CD28 were purchased from BD Biosciences. Retinoic acid and PKH were obtained from Sigma-Aldrich.

### Identification of ***ex vivo*** peripheral blood mononuclear cells

From 34 KT recipients (17 NC and 17 TCMR), PBMCs (2 × 10^5^ cells/well) were isolated from blood. In brief, cells were cultured in RPMI 1640 media. In the samples used for the *ex vivo* experiments, flow cytometry analysis was performed after the collection of blood. The cells were stained with the monoclonal antibodies: APC-CD8, streptavidin-CCR7, PE/Cy7-CD4, FITC-CD45RA, APC-CD25, PE-CD28, FITC-CD57 and FITC-CD127. Staining for chemokine receptors was performed using the following mouse monoclonal antibodies (all from BD): anti-CCR4 (1G1, IgG1), anti-CCR6 (11A9, IgG1), and anti-CCR7 (3D12, IgG2a,κ). Isotype controls were monitored non-specific binding. Cells were measured using a FACSCalibur flow cytometer and FlowJo software.

### Statistical analysis

Statistical analyses were performed using SPSS software. Continuous variables are summarized as the median with range. Independent t-tests were used for continuous variables. A non-parametric, Wilcoxon signed-rank test was used between the control and treatment groups. The chi-squared and Fisher’s exact test were used for categorical measures. A p value < 0.05 was considered significant.

## Electronic supplementary material

Supplementary information
