# Supplementary material for: Clinical significance of CCR7+CD8+ T cells in kidney transplant recipients with allograft rejection
Source: Sci Rep. 2018 Jun 11;8:8827. doi: 10.1038/s41598-018-27141-6 (PMC5995850; doi:10.1038/s41598-018-27141-6)
Supplement: Supplementary file 1 — Supplementary information [file 41598_2018_27141_MOESM1_ESM.pdf]

Clinical significance of $CCR7^+CD8^+$ T cells in kidney transplant recipients with allograft rejection

Kyoung Woon Kim[1,2], Bo-Mi Kim [1,2], Kyoung Chan Doh[1,2],

Mi-La Cho[1], Chul Woo Yang[1,2,3] and Byung Ha Chung[1,2,3]

[1]*Convergent Research Consortium for Immunologic disease*

[2]*Transplant research center*

[3]*Division of Nephrology, Department of Internal Medicine*

Seoul St. Mary's Hospital, College of Medicine, The Catholic University of Korea Seoul, Korea.

Corresponding author : Byung Ha Chung, M.D., PhD.

Department of Internal Medicine, Seoul St. Mary's Hospital

505 Banpo-Dong, Seocho-Ku, 137-040, Seoul, Korea.

Fax: +82-2-536-0323, Phone: +82-2-2258-6066

E-mail: chungbh@catholic.ac.kr

Running title: $CCR7^+CD8^+$ T cells in allograft rejection

Supplementary Figure. S1

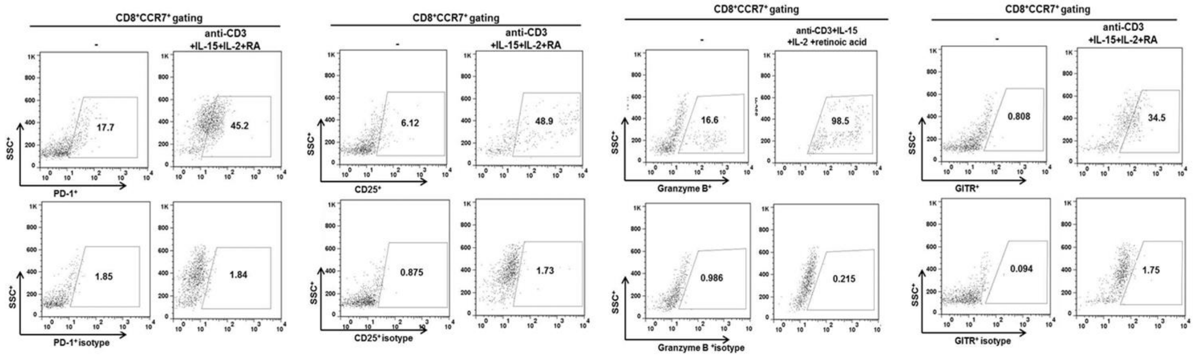

Supplementary Figure. S1. PBMCs were collected from healthy individuals, plated at 2×10^5 per well and stimulated with anti-CD3 Abs (0.1μg/ml), recombinant IL-15 (20 ng/ml), IL-2 (20 ng/ml) and retinoic acid (1 μg/ml). On day 3, cells were harvested, stained with antibodies specific for CD8, CCR7, PD-1 , CD25, Granzyme B, GITR and analyzed by flow cytometry.

Supplementary Figure. S2

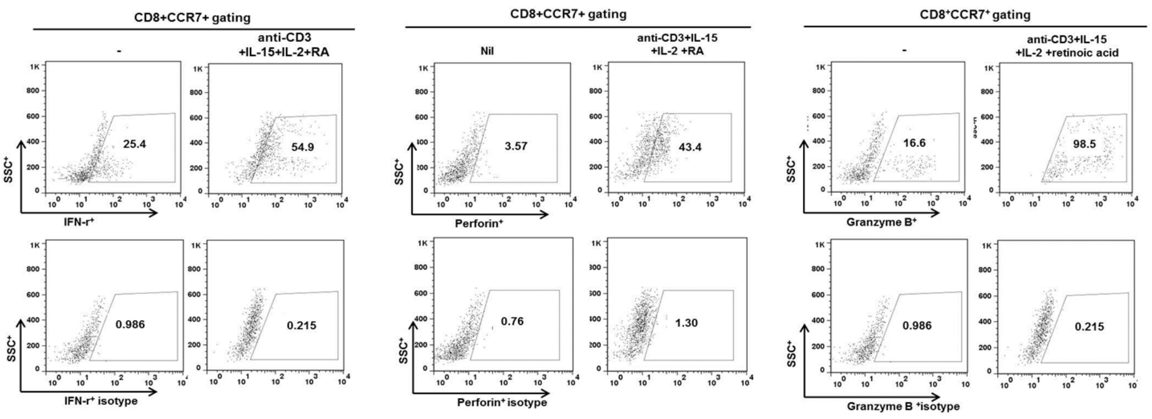

Supplementary Figure. S2. PBMCs were collected from healthy individuals, plated at 2×10^5 per well and stimulated with anti-CD3 Abs (0.1μg/ml), recombinant IL-15 (20 ng/ml), IL-2 (20 ng/ml) and retinoic acid (1 μg/ml). On day 3, cells were harvested, stained with antibodies specific for CD8, CCR7, IFN-γ , Perforin, Granzyme B and analyzed by flow cytometry.

Supplementary Figure. S3

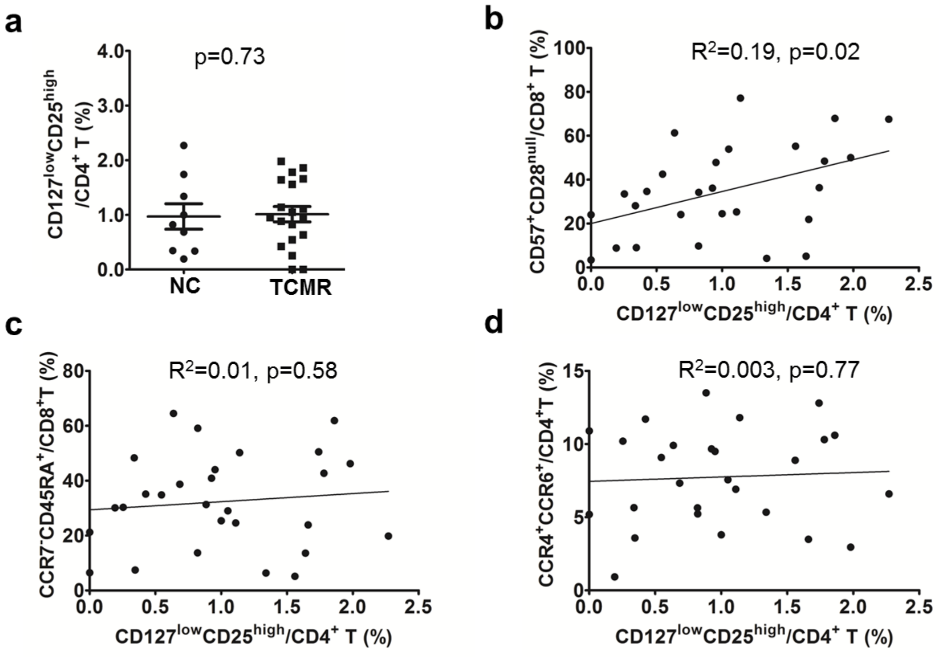

Supplementary Figure. S3. Comparison of $CD127^{low}CD25^{high}/CD4^{+}$ T cells and effector T cells ($CD57^{+}CD28^{null}/CD8^{+}$ T, $CCR7^{-}CD45RA^{+}/CD8^{+}$T and $CCR4^{+}CCR6^{+}/CD4^{+}$T cells) in peripheral blood isolated from kidney transplant recipients with or without TCMR.

Supplementary Figure. S4

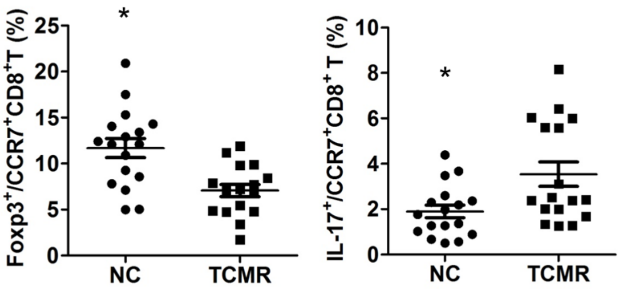

Supplementary Figure. S4. Comparison of Foxp3^{+}/CCR7^{+}CD8^{+} and IL-17^{+}/CCR7^{+}CD8^{+} in peripheral blood isolated from kidney transplant recipients with or without TCMR. *$p<0.05$ vs. TCMR.
